# Erector spinae plane block versus paravertebral block for pediatric percutaneous nephrolithotomy: interpreting procedural time and statistical non-significance

**DOI:** 10.1016/j.bjane.2026.844788

**Published:** 2026-07-01

**Authors:** Yirui Peng, Yuebing Li

**Affiliations:** Zhejiang Chinese Medical University, The Second School of Clinical Medicine, Zhejiang, China

*Dear Editor,*

We read with great interest the recent randomized trial by Nabil et al.^1^ comparing Erector Spinae Plane Block (ESPB) with thoracic Paravertebral Block (PVB) for pediatric Percutaneous Nephrolithotomy (PCNL). The authors suggest that ESPB “could be considered as an easy alternative to thoracic PVB”. The conclusion appears to place considerable emphasis on the shorter procedural time (4.37 ± 1.08 vs. 5.05 ± 1.17 minutes, p = 0.028). While we commend the authors for addressing a vital evidence gap with the first prospective trial of its kind in this population, we would like to offer a nuanced perspective on the clinical contextualization of these findings.

In a study designed as a superiority trial that found no significant difference in the primary outcome ‒ time to first rescue analgesia (15.98 ± 10.17 vs.18.18 ± 9.18 hours, p = 0.464) ‒ emphasizing procedural speed as a decisive factor may inadvertently oversimplify the complex priorities of pediatric regional anesthesia. Reliability of analgesia and established safety profiles typically carry greater clinical weight than marginal efficiency gains.

The methodology employed by Nabil et al. is robust, utilizing assessor-blinding, standardized anesthetic protocols, and the validated FLACC scale assessment.^2^ The finding that no statistically significant difference was observed in analgesic efficacy ‒ supported by overlapping 95% Confidence Intervals and similar intraoperative hemodynamics ‒ suggests that a clinical advantage of one technique over the other was not demonstrated in this study. However, the 40-second time difference, while statistically significant, represents less than 1% of the total operative time (approximately 85 minutes), which likely limits its practical relevance for operating room throughput. Furthermore, as the study was powered to detect differences in analgesia rather than procedural duration, the p = 0.028 finding should be interpreted with caution in the absence of a dedicated sample size calculation for this secondary outcome.

Beyond statistics, certain mechanistic distinctions warrant consideration. PVB involves the direct deposition of local anesthetic into the paravertebral space, providing reliable blockade of spinal nerves and sympathetic fibers at T10‒L1, which is critical for managing the visceral pain associated with renal surgery. In contrast, ESPB relies on indirect diffusion through the costotransverse foramina. Anatomical studies suggest this pathway may yield more variable dermatomal coverage, particularly in pediatric patients whose fascial anatomy differs from that of adults.^3^

Regarding safety, a cohort of 55 patients lacks the statistical power to detect rare but serious complications such as pneumothorax or neuraxial spread. While ESPB is often favored for its theoretical safety due to its superficial nature, the pediatric evidence base for PVB is currently more robust. For instance, an observational study of 871 thoracic PVB cases demonstrated reassuringly low complication rates, whereas the pediatric safety database for ESPB remains relatively limited.^4^

Finally, we note that the blocks were performed by a single, highly experienced practitioner. The exceptionally low block failure rate (one per group) suggests that both techniques are highly reliable when performed with expertise, perhaps diminishing the perceived advantage of “ease of use” in settings where technical proficiency is already established.

In conclusion, these data demonstrate a lack of clear analgesic superiority for ESPB, suggesting it should be viewed as a comparable alternative. Optimal block selection should likely remain a tailored decision based on institutional expertise, patient anatomy, and specific surgical requirements. We endorse the authors' call for larger trials, which might ideally be powered to evaluate complication rates and cost-effectiveness.

Sincerely.

## Declaration of generative AI and AI-assisted technologies in the manuscript preparation process

During the preparation of this manuscript, the authors used Gemini for language editing and grammatical clarity only. The authors reviewed and edited the content as needed and take full responsibility for the content of the publication.

## Data availability statement

No new data were generated or analyzed in this article. Data sharing is not applicable to this article.

## Authors’ contributions

Yirui Peng: Conceptualization; data curation; formal analysis; investigation, methodology; writing-original draft preparation.

Yuebing Li: Critical revision for important intellectual content, and final approval.

## Funding

None.

## Conflicts of interest

The authors declare no conflicts of interest.
